# P-390. Persistence and Adherence of People With HIV (PWH) and Mental Health Disorder Diagnosis Who Restart Antiretroviral Therapy (ART)

**DOI:** 10.1093/ofid/ofaf695.607

**Published:** 2026-01-11

**Authors:** Travis Lim, Amanda Kong, Mary J Christoph, Uche Mordi, Jacqueline Lucia, Daisha Joseph, Gulce Askin, Daniela Yucuma, Neia Prata Menezes

**Affiliations:** Gilead Sciences, Inc., Washington, DC; Aetion, New York, New York; Gilead Sciences, Inc., Washington, DC; Gilead Sciences, Inc., Washington, DC; Aetion, New York, New York; Aetion, New York, New York; Aetion, New York, New York; Aetion, New York, New York; Gilead Sciences, Inc., Washington, DC

## Abstract

**Background:**

Nearly half of PWH on ART have a mental health or substance use disorder (MH/SUD) comorbidity and thus have a higher risk of treatment interruptions (TI). TI can lead to viremia, drug resistance, clinical progression, and transmission of HIV. We compared persistence and adherence by regimen for PWH with MH/SUD resuming ART after a gap in therapy.

**Figure:**
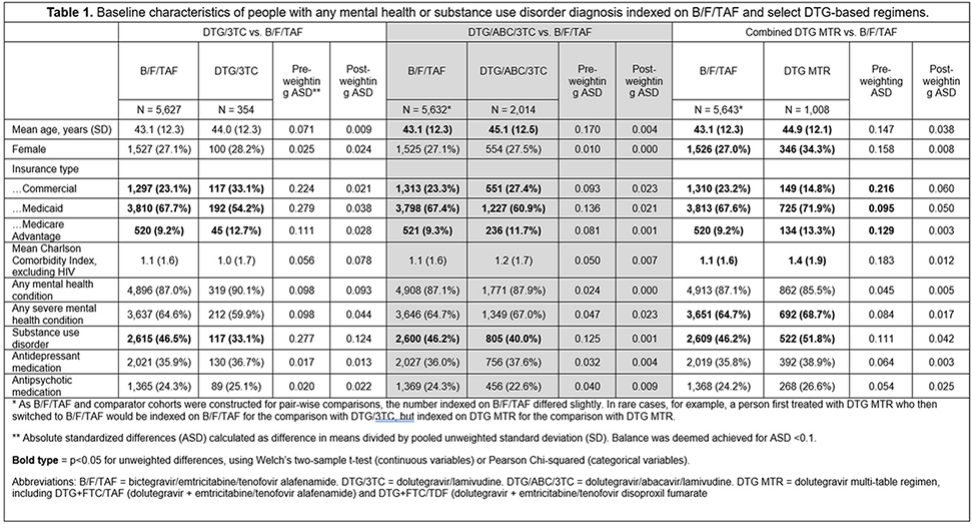

**Figure:**
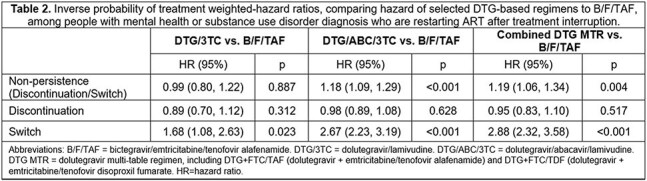

**Methods:**

We analyzed pharmacy and medical claims data from HealthVerity Marketplace from January 2015-February 2024. PWH aged ≥18 years old restarting one of the following index regimens after a ≥90 day gap in therapy were identified: B/F/TAF, DTG/3TC, DTG/ABC/3TC, or DTG-based multi-tablet regimen (MTR; DTG+TDF/FTC or DTG+TAF/FTC). Continuous enrollment was required for ≥ 365 days pre-restart and ≥ 90 days post-restart. We calculated non-persistence as a discontinuation (medication gap of 91+ days) or regimen switch. Adherence was measured as the proportion of days covered while persistent. We fit inverse probability of treatment weighted (IPTW) Cox models to calculate pair-wise hazard ratios (HR) of non-persistence outcomes, with censoring for disenrollment, evidence of pregnancy, or end of study, comparing B/F/TAF to each other regimen.

**Results:**

20,623 unique individuals were indexed restarting the same ART after a TI > 90 days. Of these, 8,953 (43.4%) had any mental health diagnosis, including 5,857 (28.4%) with severe mental health disorder and 4,015 (19.5%) with substance use disorder. The proportion of PWH insured by Medicaid differed significantly across ARTs, ranging from 71.9% of PWH taking DTG MTRs and 54.2% of those on DTG/3TC. After IPTW for restarters with MH/SUD, balance was achieved on baseline characteristics (Table 1). Upon re-starting, compared to individuals on B/F/TAF, the hazard of subsequent non-persistence was higher for DTG/ABC/3TC and DTG MTR, but similar for DTG/3TC (Table 2). Compared to B/F/TAF, the hazard of switching was higher for all regimens, including DTG/3TC (Table 2). Adherence was similar by regimen (PDC range 78.7–80.2%).

**Conclusion:**

MH/SUD are prevalent among PWH with TI resuming ART. PWH with MH/SUD disorders on B/F/TAF were more persistent compared to DTG/ABC/3TC and DTG MTR, and less likely to switch therapy vs. those on DTG/3TC.

**Disclosures:**

Travis Lim, MSc, DrPH, Gilead Sciences, Inc.: Employee|Gilead Sciences, Inc.: Stocks/Bonds (Public Company) Amanda Kong, DrPH, Aetion: Employee|Aetion: Stocks/Bonds (Private Company) Mary J. Christoph, PhD, MPH, AstraZeneca: Advisor/Consultant|AstraZeneca: Employee|AstraZeneca: Stocks/Bonds (Public Company)|Gilead Sciences, Inc.: Employee|Gilead Sciences, Inc.: Stocks/Bonds (Public Company) Uche Mordi, PharmD, MS, Gilead Sciences, Inc.: Stocks/Bonds (Public Company) Jacqueline Lucia, BS, Aetion: Employee|Aetion: Stocks/Bonds (Private Company) Gulce Askin, MPH, Aetion: Employee|Aetion: Stocks/Bonds (Private Company) Daniela Yucuma, MD, MPH, Aetion: Employee Neia Prata Menezes, PhD, Gilead Sciences, Inc.: Stocks/Bonds (Private Company)

